# Overexpression of the *UGT73C6 *alters brassinosteroid glucoside formation in *Arabidopsis thaliana*

**DOI:** 10.1186/1471-2229-11-51

**Published:** 2011-03-24

**Authors:** Sigrid Husar, Franz Berthiller, Shozo Fujioka, Wilfried Rozhon, Mamoona Khan, Florian Kalaivanan, Luisa Elias, Gillian S Higgins, Yi Li, Rainer Schuhmacher, Rudolf Krska, Hideharu Seto, Fabian E Vaistij, Dianna Bowles, Brigitte Poppenberger

**Affiliations:** 1 Max F. Perutz Laboratories, University of Vienna, Dr. Bohr-Gasse 9, 1030 Vienna, Austria; 2 Center for Analytical Chemistry, Department of Agrobiotechnology, University of Natural Resources and Life Sciences, Konrad Lorenz Straße 20, 3430 Tulln, Austria; 3 RIKEN Advanced Science Institute, Wako-shi, Saitama 351-0198, Japan; 4 Center for Novel Agricultural Products, Department of Biology, University of York, York YO10 5DD, UK

**Keywords:** arabidopsis, brassinosteroids, glycosylation, homeostasis, malonylation, steroids

## Abstract

**Background:**

Brassinosteroids (BRs) are signaling molecules that play essential roles in the spatial regulation of plant growth and development. In contrast to other plant hormones BRs act locally, close to the sites of their synthesis, and thus homeostatic mechanisms must operate at the cellular level to equilibrate BR concentrations. Whilst it is recognized that levels of bioactive BRs are likely adjusted by controlling the relative rates of biosynthesis and by catabolism, few factors, which participate in these regulatory events, have as yet been identified. Previously we have shown that the UDP-glycosyltransferase UGT73C5 of *Arabidopsis thaliana *catalyzes 23-*O*-glucosylation of BRs and that glucosylation renders BRs inactive. This study identifies the closest homologue of UGT73C5, UGT73C6, as an enzyme that is also able to glucosylate BRs *in planta*.

**Results:**

In a candidate gene approach, in which homologues of UGT73C5 were screened for their potential to induce BR deficiency when over-expressed in plants, UGT73C6 was identified as an enzyme that can glucosylate the BRs CS and BL at their 23-*O*-positions *in planta*. GUS reporter analysis indicates that *UGT73C6 *shows over-lapping, but also distinct expression patterns with *UGT73C5 *and YFP reporter data suggests that at the cellular level, both UGTs localize to the cytoplasm and to the nucleus. A liquid chromatography high-resolution mass spectrometry method for BR metabolite analysis was developed and applied to determine the kinetics of formation and the catabolic fate of BR-23-*O*-glucosides in wild type and *UGT73C5 *and *UGT73C6 *over-expression lines. This approach identified novel BR catabolites, which are considered to be BR-malonylglucosides, and provided first evidence indicating that glucosylation protects BRs from cellular removal. The physiological significance of BR glucosylation, and the possible role of UGT73C6 as a regulatory factor in this process are discussed in light of the results presented.

**Conclusion:**

The present study generates essential knowledge and molecular and biochemical tools, that will allow for the verification of a potential physiological role of UGT73C6 in BR glucosylation and will facilitate the investigation of the functional significance of BR glucoside formation in plants.

## Background

Brassinosteroids (BRs) are a family of steroid hormones that regulate cell division and cell elongation in plants and participate in the control of growth and development [1]. BRs are synthesized from the sterol campesterol, which is modified by a cascade of hydroxylation and oxidation reactions to yield the biologically active BRs castasterone (CS) and brassinolide (BL) [2]. CS and BL bioactivity is conferred by their ability to bind to the BR-receptor BRI1 [3], which initiates a phosphorylation-dependent signal transduction cascade leading to nuclear acquisition of transcription factors that regulate the expression of BR-responsive genes [4].

Whereas the last decade has seen rapid progress in the identification and characterization of factors, which control BR biosynthesis and participate in BR signal transduction, fewer advances were made in identifying proteins, which directly regulate BR cellular homeostasis. Different homeostatic mechanisms are thought to operate to maintain a BR equilibrium, including the feedback inhibition of BR production [5]. In addition, catabolic inactivation is also considered to play a role in the regulation of bioactive BR levels [2]. CS and BL are catabolically altered or conjugated, with some modifications yielding inactive products. Hydroxylation is one means of catabolic inactivation and is catalyzed by the *Arabidopsis thaliana *cytochrome P450 monooxygenase BAS1 [6]. Another class of BR conjugates, which are inactive, are glucosides. CS and BL were found to be glucosylated at different positions in feeding studies, with species-specific variations in BR-glucoside profiles [7]. In *A. thaliana *the hydroxyl groups C-2 and C-23 of CS and BL were identified as target sites for an attachment of glucose [7,8]. Whilst enzymes mediating C-2 glucosylation of BRs are still unknown, we could previously show that 23-*O*-glucosylation of CS and BL in *A. thaliana *is catalyzed by UGT73C5, a UDP-glycosyltransferase (UGT) [8]. An increase in BR-23-*O*-glucosylation activity in *UGT73C5 *over-expressing plants correlated with reduced levels of typhasterol (TY), 6-deoxocastasterone (6-deoxoCS) and CS and with BR-deficient phenotypes, showing that 23-*O*-glucosylation reduces BR bioactivity [8].

UGTs are glycosyltransferases (GTs) of family 1 in the CAZy classification of carbohydrate-active enzymes [9] and catalyze the transfer of glycosyl donor groups to small molecule acceptors, which include secondary metabolites, biotic and abiotic toxins and plant hormones [10,11]. UGTs are regio- and stereo-selective, but are often capable *in vitro *of recognizing common features on multiple substrates [12]. Moreover, from studies in the multigene family of UGTs in *A. thaliana*, it has become clear that *in vitro *a single substrate may be accepted by many individuals of the family [11,12]. UGT73C5 has evolved from UGT subfamily 73C [13], which consists of seven UGTs, six of which are clustered in a tandem repeat, are highly similar in their sequences and are promiscuous in their substrate acceptance *in vitro*. For example, UGT73C6 has been reported as a flavonoid-7-*O*-glycosyltransferase [14] and is *in vitro *also capable of conjugating hydroxycoumarins [12], the isoflavone daidzein, the stilbene *trans*-resveratrol [15], the xenobiotics hydroxylaminodinitrotoluene and aminodinitrotoluene [16], as well as in yeast the fungal toxin zearalenone [17]. Whereas activities of UGT73C subfamily members have been analyzed against various substrates *in vitro *[13,15,16,18] the *in planta *substrate specificities and the physiological roles of these UGTs are, with the exception of UGT73C5, as yet little defined.

This study extends and completes the analysis of the UGT73C cluster in regard to the potential of its members to glucosylate BRs and identifies UGT73C6 as a second UGT, which can accept BRs as substrates *in planta*. It is shown that over-expression of *UGT73C6 *induces BR-deficient phenotypes, whereas an over-expression of the *UGTs 73C1, 73C2, 73C3 *and *73C4 *does not cause such effects. BR metabolite profiles and BR glucosylation activity analyses provide evidence that UGT73C6 can catalyze CS and BL 23-*O*-glucosylation *in planta*. This work also introduces a liquid chromatography high-resolution mass spectrometry (LC-HRMS) method, developed for the detection of BR metabolites, and used as a tool to determine the kinetics of BR-23-*O*-glucoside formation in wild type, *UGT73C5oe *and *UGT73C6oe *plants. The analysis uncovered the existence of novel BR catabolites, which are considered to be BR-malonylglucosides. LC-HRMS of the kinetics of BL uptake and catabolism in *UGT73C5oe *and *UGT73C6oe *lines as compared to wild type provided first indications that glucosylation protects BL from cellular removal.

## Results

### Over-expression of *UGT73C6 *results in BR deficiency in *A. thaliana*

**Figure 1:**
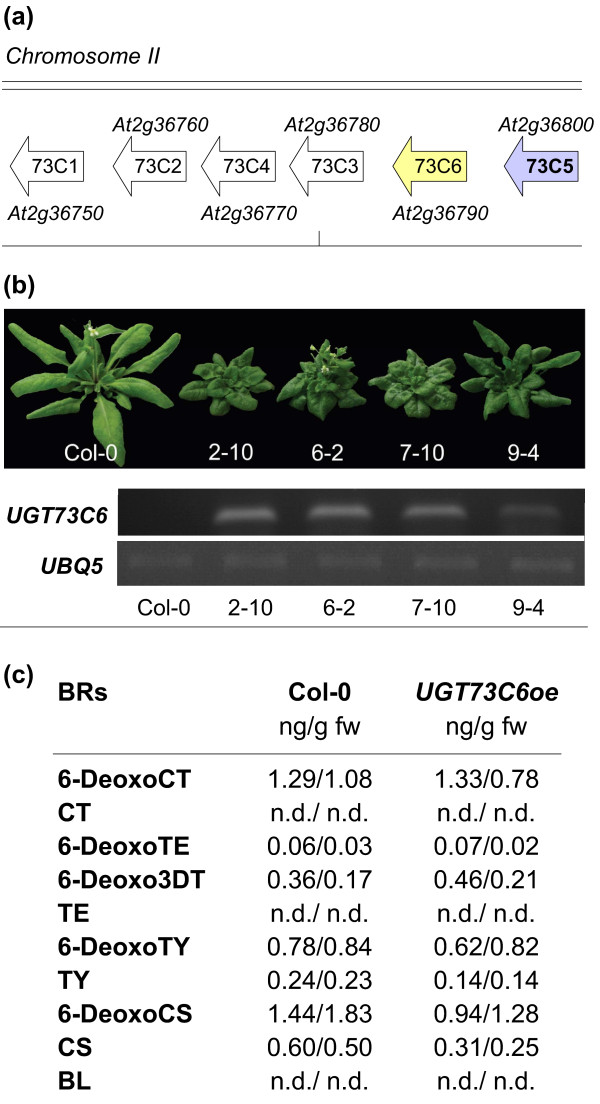
**Characterization of *UGT73C6 *over-expressing lines**. (a) Illustration of the *UGT73C *gene cluster. (b) Adult phenotypes of independent transgenic lines expressing a *35S_pro_:UGT73C6 *construct as compared to wild type Col-0, grown for 4 weeks in long-day conditions (16 hrs 80-100 μmol·m^-2^·s^-1 ^white light/8 hrs dark) at 21 ± 2°C. Semi-quantitative RT PCR analysis of *UGT73C6 *transcript levels in the lines whose phenotype is shown. *UBQ5 *served as an internal control. (c) BR contents in *UGT73C6oe *plants as compared to wild type. BR contents were quantified by GC-MS in two independent experiments in which aerial tissues of *A. thaliana *plants, grown in the same conditions as in (a) for 24 d, were compared. BR levels in ng/g fw are shown. nd, not detected (below the limit of detection).

*UGT73C5 *is a member of UGT subfamily 73C, which is comprised of seven genes, six of which are clustered in a tandem repeat on Chromosome 2 (Figure 1a). The genes of the cluster are highly similar to each other, suggesting that they have evolved from a gene duplication from one ancestral gene and may therefore have related enzymatic properties [19].

Earlier work, which focused on *UGT73C5*, had demonstrated that constitutive over-expression led to strong BR-deficient phenotypes [8]. Thus, it was of interest to investigate the phenotypic consequences of over-expressing other members of the *UGT73C *gene cluster. 15 to 25 independent transgenic lines expressing each of the *UGTs 73C1*, *73C2*, *73C3*, *73C4 *and *73C6 *under control of the constitutive *Cauliflower Mosaic Virus 35 S *(*CaMV35S*) promoter were generated and analyzed for steady-state levels of transcripts using semi-quantitative RT-PCRs. 3 to 5 lines with high expression levels were then chosen for each UGT to assess effects on plant growth and development. Whereas plants over-expressing the *UGT73C1, UGT73C2, UGT73C3 *and *UGT73C4 *did not show any obvious morphological phenotypes (data not shown), an elevated expression of *UGT73C6 *resulted in drastic growth defects indicative for BR deficiency. As shown in Figure 1b*UGT73C6 *over-expressing (*UGT73C6oe*) plants were characterized by dark-green leaves with short petioles and a cabbage-like morphology, delayed flowering and senescence and reduced fertility; these phenotypes correlated in severity with the amounts of *UGT73C6 *transcript present.

To verify if the phenotypic indications for BR deficiency were correlated with changes in endogenous BR levels, BR amounts were analyzed in aerial plant parts of a line with strong *UGT73C6 *expression (*UGT73C6oe/2-10*) and compared to wild type by GC-MS in two independent biological experiments. The results are illustrated in Figure 1c and show that concentrations of TY, 6-deoxoCS and CS were reduced in *UGT73C6oe *plants. BL was below the limit of detection in both *UGT73C6oe *and wild type plants.

Taken together these results show that over-expression of *UGT73C6 *induced phenotypes indicative of impaired BR action in *A. thaliana*, which correlated with reduced levels of late pathway intermediates of BR biosynthesis.

### UGT73C6 catalyzes 23-*O*-glucosylation of CS and BL *in planta*

UGT73C6 has previously been characterized as a UDP-glucose:flavonol-3-*O*-glycoside-7-*O*-glucosyltransferase, based on a decrease in quercetin-3-*O*-rhamnoside-7-*O*-glucoside accumulation in flowers of a *UGT73C6 knock-out *(*UGT73C6ko*) line and a respective catalytic activity *in vitro *[14]. To investigate the possibility that UGT73C6, in addition to its role in quercetin-3-*O*-rhamnoside glucosylation, can also catalyze BR glucosylation *in planta*, it was anticipated to analyze BR glucoside formation in plants altered in *UGT73C6 *expression. For this purpose a LC-HRMS method was developed, which is outlined in the experimental procedures section. As reference standards CS-2-*O*-glucoside (CS-2Glc), CS-3-*O*-glucoside (CS-3Glc), CS-22-*O*-glucoside (CS-22Glc), CS-23-*O*-glucoside (CS-23Glc), BL-2-*O*-glucoside (BL-2Glc), BL-3-*O*-glucoside (BL-3Glc), BL-22-*O*-glucoside (BL-22Glc) and BL-23-*O*-glucoside (BL-23Glc) were used. The identification was based on retention times and mass spectra, by direct comparison of standards and metabolites. Recovery rates for all measured analytes were between 83% and 93% (except for CS with 63% recovery), with a repeatability ranging from 1.8% to 3.9%.

Preliminary experiments showed that, in accordance with previous studies [7,8], endogenous BR glucosides were below the limit of detection in untreated plants, also with the newly developed LC-HRMS method. Thus BR glucoside formation was investigated in plants treated with CS or BL. Ten-day-old light-grown seedlings of wild type and *UGT73C5oe *plants, as well as *UGT73C6oe *and *UGT73C6ko *plants, were incubated in media containing either CS or BL for 48 hrs and metabolites formed were measured by LC-HRMS. The results of the feeding studies showed that in plants over-expressing the *UGT73C6*, in correspondence with plants over-expressing *UGT73C5*, CS-23Glc and BL-23Glc formation was strongly increased (Table 1) whereas CS-2Glc and BL-2Glc levels appeared unaltered (data not shown). In seedlings of *UGT73C6ko *plants no statistically significant differences in BR glucosylation activities to wild type were found. Interestingly CS-23Glc and BL-23Glc were not only present in plant extracts, but were also detected in the media, in which the plants had been incubated for the feeding studies (Table 1).

**Table 1:** Glucosides of BRs measured in seedlings of *UGT73C5oe*, *UGT73C6oe *and wild type used in BR feeding studies.

	Plant line	Plant extracts ng/g Fw	Media ng
**BL-23Glc**	Wild type	310.8 ± 77.3	4.4 ± 2.2
	*UGT73C5oe*	1402.5 ± 361.7	46.0 ± 18.4
	*UGT73C6oe*	1489.1 ± 103.5	23.6 ± 3.5
	*UGT73C6ko*	428.2 ± 68.6	1.1 ± 0.4
			
**CS-23Glc**	Wild type	34.0 ± 8.4	5.3 ± 3.1
	*UGT73C5oe*	153.7 ± 61.9	37.1 ± 7.9
	*UGT73C6oe*	154.6 ± 37.5	43.2 ± 10.9
	*UGT73C6ko*	47.1 ± 9.6	0.7 ± 0.6

Standard deviation of three independent biological experiments is shown.

Therefore the results are consistent with the hypothesis that UGT73C6 can catalyze 23-*O*-glucosylation of CS and BL *in planta*.

### *UGT73C6 *promoter GUS activity is developmentally regulated

**Figure 2:**
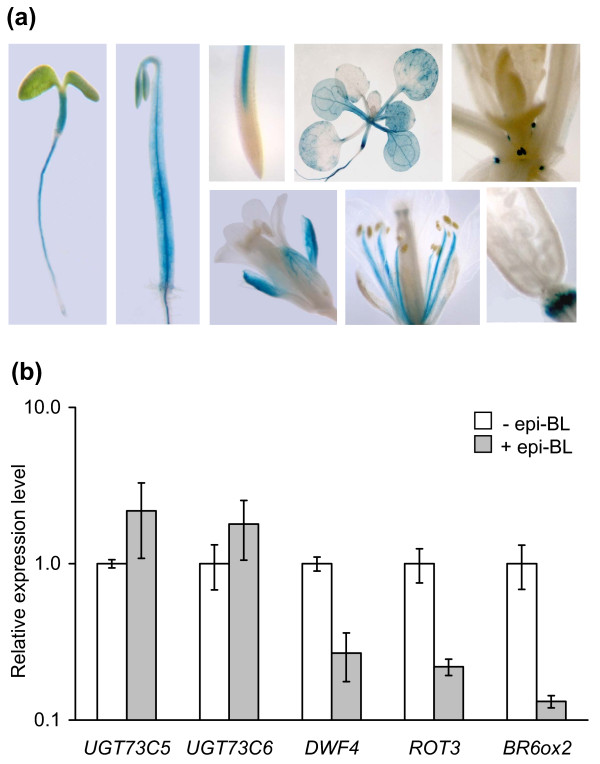
***UGT73C6_pro_:GUS *expression is present in all organs and is developmentally regulated**. (a) A homozygous line expressing a *UGT73C6 *promoter GUS fusion, that showed a characteristic staining pattern, was chosen for histochemical analysis of *UGT73C6_pro_:GUS *expression in different organs and developmental stages. (b) Response of *UGT73C5*, *UGT73C6*, *DWF4*, *ROT3 *and *BR6ox2 *expression in eight-day-old whole seedlings to external application of 1 μM 24-epiBL. *GAPC2 *was used for standardization. The mean and standard deviation of three biological replicates is shown. *UGT73C5 *and *UGT73C6 *expression levels are not statistically significantly altered (t-test p-value > 0.05) in treated versus untreated samples, while the expression of *DWF4*, *ROT3 *and *BR6ox2 *is significantly repressed (t-test p-value < 0.01) by 1 μM of 24-epiBL.

To analyze the promoter activity of *UGT73C6 *in different tissues and developmental stages a GUS reporter lines was constructed in which the GUS gene was expressed under control of the *UGT73C6 *promoter (*UGT73C6_pro_:GUS*). Histochemical analysis of GUS expression in these lines revealed that the *UGT73C6 *promoter was active in a number of different cell types and was developmentally regulated (Figure 2). Early in development, GUS reporter expression was similar to that previously observed in *UGT73C5_pro_:GUS *plants [20]: a pronounced staining in the vasculature of roots and hypocotyls of young seedlings, both when grown in the light and when incubated in the dark. However, as opposed to *UGT73C5_pro_:GUS *plants GUS reporter expression in *UGT73C6_pro_:GUS *seedlings was not observed in epidermal cells of the root elongation zone. Later in seedling development, in analogy to *UGT73C5_pro_:GUS *the *UGT73C6_pro_:GUS *reporter was still active in roots and hypocotyls and moreover was also expressed in cotyledons and true leaves. In contrast to *UGT73C5_pro_:GUS*, *UGT73C6_pro_:GUS *was in addition strongly expressed in stipules. In flowers *UGT73C6_pro_:GUS *activity was present in sepals and in the stamen filaments. Similar to *UGT73C5*, *UGT73C6 *promoter expression was also detected in abscission zones.

In summary there is evidence that the *UGT73C6 *promoter is subjected to developmental regulation and that it is active in tissues, in which BRs are also known to act. It is worth noting that, when analyzed, the *UGT73C6_pro_:GUS *reporter was not found to be responsive to externally applied BR (data not shown). To verify this result quantitative real-time PCR (qPCR) analysis of 8-day old seedlings of wild type, treated with 24-epiBL for 24 hrs, was performed. The result showed that in this developmental stage, at 24 hrs post application, 24-epiBL had little effect on *UGT73C6 *expression on a whole seedling level (Figure 2b). In these conditions also *UGT73C5 *expression was not significantly altered, whereas *DWF4*, *ROT3 *and *BR6ox2*, genes that are repressed by BR application [5], were significantly decreased in their expression.

### Subcellular localization of *UGT73C6 *expression

**Figure 3:**
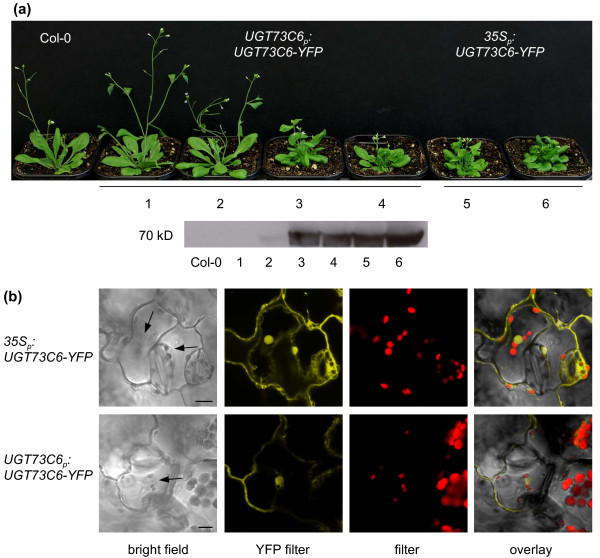
**UGT73C6-YFP reporter generation and analysis**. (a) Top: Adult phenotypes of independent transgenic lines expressing either a *UGT73C6_pro _:UGT73C6-YFP *construct (center) or a *35S_pro_:UGT73C6-YFP *construct (right) as compared to wild type (wt), grown for 4 weeks in long-day conditions (16 hrs 80-100 μmol·m^-2^·s^-1 ^white light/8 hrs dark) at 21 ± 2°C. Bottom: Western blot analysis of UGT73C6-YFP protein levels in 2-week-old seedlings of the lines whose phenotype is shown above, using an anti-GFP antibody. (b) Representative YFP expression pattern of UGT73C6-YFP analyzed in leaves of eleven-day-old seedlings of line *35S_pro_:UGT73C6-YFP/4 *and line *UGT73C6_pro_:UGT73C6-YFP/5 *by fluorescence microscopy. The scale bars represent 10 μm.

To investigate the cellular sites of UGT73C6 protein localization plants expressing *UGT73C6-YFP *reporter constructs were generated. For this purpose two vectors were cloned: one in which the YFP-tagged coding sequence of *UGT73C6 *was placed down-stream of its own promoter (*UGT73C6_pro_:UGT73C6-YFP*), and another one in which the *UGT73C6-YFP *fusion was driven by the *CaMV35 S *promoter. *A. thaliana *plants stably expressing the two constructs were generated and YFP expression levels were assessed in seedlings of homozygous lines using Western blot analysis. The results are illustrated in Figure 3a and show that lines 3, 4, 5 and 6 expressed *UGT73C6-YFP *to high levels; these lines also showed BR-deficient phenotypes, indicating that the UGT73C6-YFP fusion was active.

Imaging of *YFP *expression in seedlings of *35S_pro_:UGT73C6-YFP *and *UGT73C6_pro_:UGT73C6-YFP *lines using confocal microscopy revealed that UGT73C6-YFP was localized in the cytoplasm, as well as in the nucleus (Figure 3b). As expected fluorescence in *35S_pro_:UGT73C6-YFP *was stronger than in *UGT73C6_pro_:UGT73C6-YFP *lines, but showed an identical localization pattern. *35S_pro_:UGT73C5-YFP *showed the same subcellular localization pattern as *35S_pro_:UGT73C6-YFP *(data not shown).

**Figure 4:**
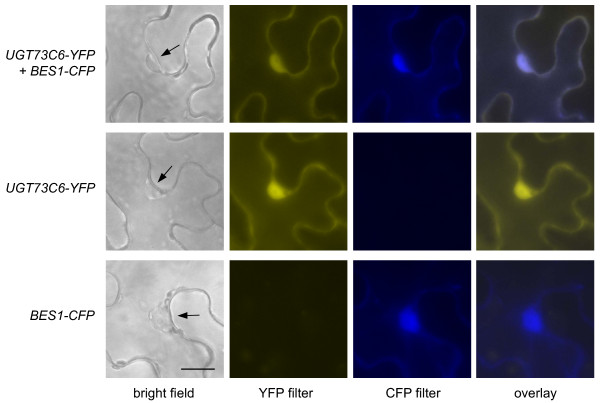
**Transient co-localization studies of UGT73C6-YFP and BES1-CFP in tobacco**. UGT73C6-YFP and BES1-CFP were transiently co-expressed in leaves of *Nicotiana benthamiana *and localization was examined by fluorescence microscopy. All pictures were taken with the same magnification. The scale bar represents 10 μm.

To verify the nuclear localization of UGT73C6-YFP the reporter was transiently co-expressed with a CFP fusion of BES1, a protein that is known to localize predominantly to the nucleus [4], in tobacco. The result showed that UGT73C6-YFP co-localized with BES1-CFP (Figure 4) providing evidence that the UGT73C6-YFP reporter, in addition to the cytoplasm also localizes to the nucleus.

### Kinetics of BL-23-*O*-glucoside formation

**Figure 5:**
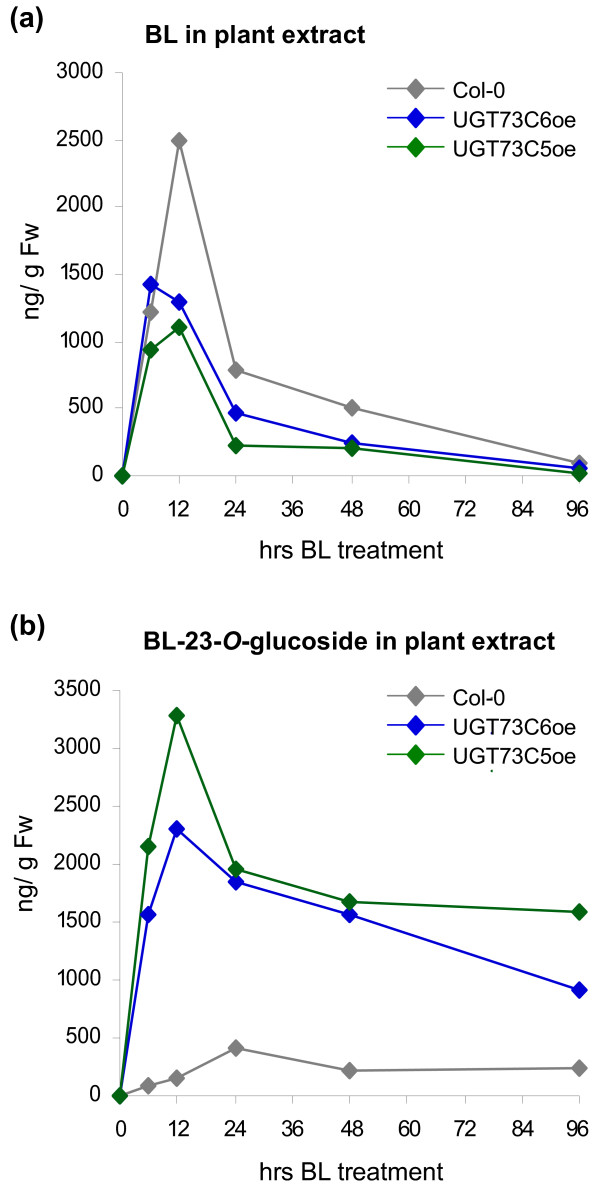
**BL and BL-23Glc levels formed in seedlings of *A. thaliana *used in BL feeding studies over time, analyzed by LC-HRMS**. eleven-day-old seedlings were incubated for the indicated periods of time in ATS media supplemented with 30 μg BL, and BL contents were quantified from plant extracts by LC-HRMS analysis. (a) BL and (b) BL-23Glc levels are shown in ng/g Fw.

To investigate the conversion of BL into BL-23Glc in *UGT73C5oe *and *UGT73C6oe *plants over time, a time-course feeding study was initiated. Eleven-day-old seedlings of wild type Col-0, *UGT73C5oe *and *UGT73C6oe *were incubated with 1 μg/ml (2.1 μM) of BL. Samples were harvested in a time-course manner and BL-23Glc formation was determined in tissue extracts by LC-HRMS. As shown in Figure 5a BL was rapidly incorporated, as evidenced by a strong increase in endogenous BL amounts following BL application. In wild type seedlings BL levels increased rapidly for 12 hrs following BL application, before they started to decline. BL levels in *UGT73C5oe *and *UGT73C6oe *also increased for approximately 12 hrs post application of BL, however BL amounts only reached about 50% of the levels, which were accumulated in wild type (Figure 5a). 96 hrs post application, BL levels in both wild type and *UGT73C5oe *and *UGT73C6oe *lines had dropped to levels below the limit of detection.

BL-23Glc formation in wild type seedlings slowly increased for 24 hrs, before BL-23Glc amounts started to decrease. In *UGT73C5oe *and *UGT73C6oe *plants the concentration of BL-23Glc strongly increased for approximately 12 hrs, reaching amounts which were approximately 10-fold higher, than those measured in wild type (Figure 5b).

In summary exogenously applied BL was rapidly incorporate by both wild type and *UGT73C5oe *and *UGT73C6oe *plants and was thereafter efficiently removed. In contrast, following its formation, BL-23Glc was maintained at elevated levels in plant tissues.

### Catabolic fate of BR-23-*O*-glucosides

**Figure 6:**
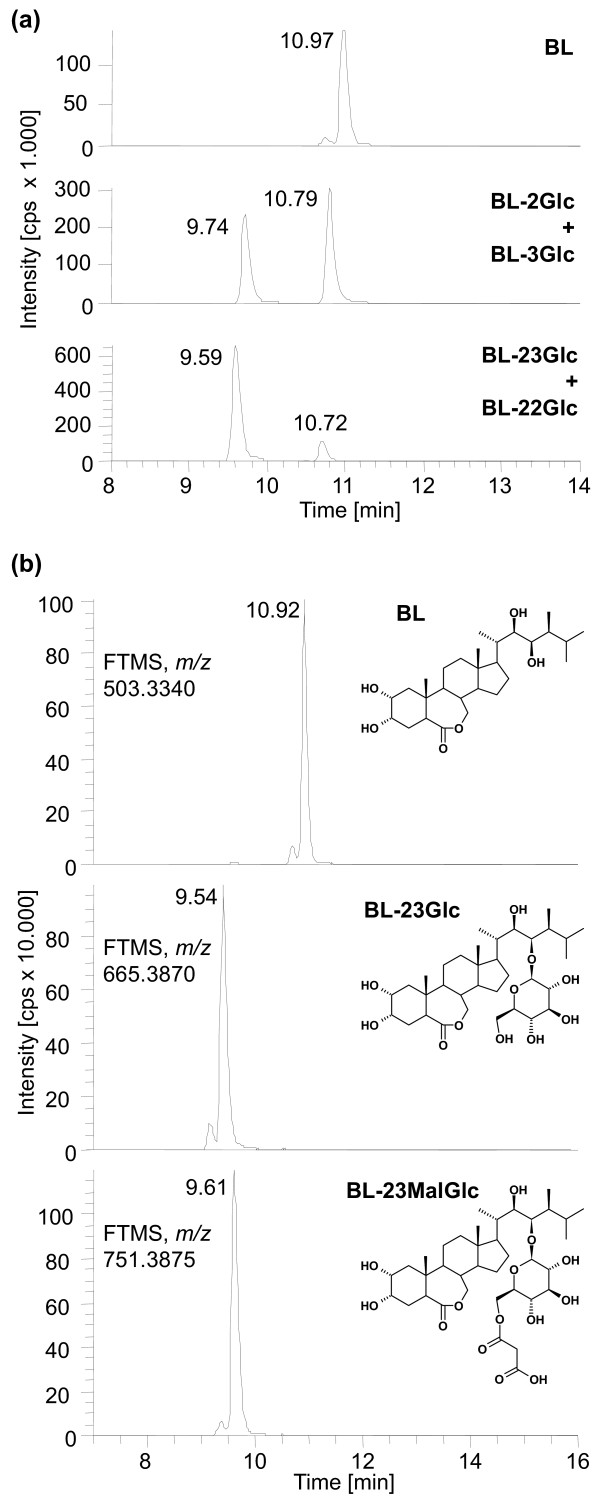
**Identification of a novel BL-Glc catabolite**. HR-LCMS analysis was employed to identify glucosides formed in BL feeding experiments of seedlings over-expressing *UGT73C5*. (a) HR-LCMS mass chromatograms of authentic BL-*O*-glucoside standards. (b) HR-LCMS mass chromatograms of metabolites formed in *UGT73C5oe *seedlings. Theoretical masses of sodium adducts and predicted structures are shown. The position of the malonylgroup in the putative BL-23MalGlc is not certain.

The decrease of BL-23Glc levels in plant tissues, starting at 12 hrs post application of BL in *UGT73C5oe *and *UGT73C6oe *seedlings and at 24 hrs in wild type, indicated that the BL-23Glc formed was either immobilized, degraded or was further modified to yield products, which escaped detection. Also, previously it had been shown that in BL-feeding studies of wild type *A. thaliana*, an initial increase in BL-23Glc formation was followed by a decrease, indicating a further metabolization [7]. Thus it was of interest to investigate the catabolic fate of externally applied CS and BL. LC-HRMS was used to analyze BR conjugates in seedlings of wild type, *UGT73C5oe *and *UGT73C6oe *plants, following 48 hrs of incubation with either CS or BL. In addition to significant amounts of BR-23Glc, minor peaks corresponding to BR-2Glc, BR-sulfate and BR-hydroxide were found. Moreover, very interestingly, a previously unknown substance with a mass of *m/z *751.3877 eluted at 9.61 min (compared to 9.54 min of BL-23Glc), in seemingly high abundance, from the column (Figure 6). According to accurate mass measurements the compound was tentatively identified as BL-malonylglucoside (BL-MalGlc). The theoretical mass of the sodium adduct of this substance is 751.3875 (0.2 ppm deviation), the only possible sum formula is C_37_H_60_O_14 _(subtracting the sodium adduct; nitrogen rule applied; max. 1 ppm mass deviation; max. 10 nitrogen, 30 oxygen, 100 carbon and 200 hydrogen atoms). As malonylglucosides are formed from glucosides it is highly likely that the compound is BL-23-*O*-malonylglucoside (BL-23MalGlc).

**Figure 7:**
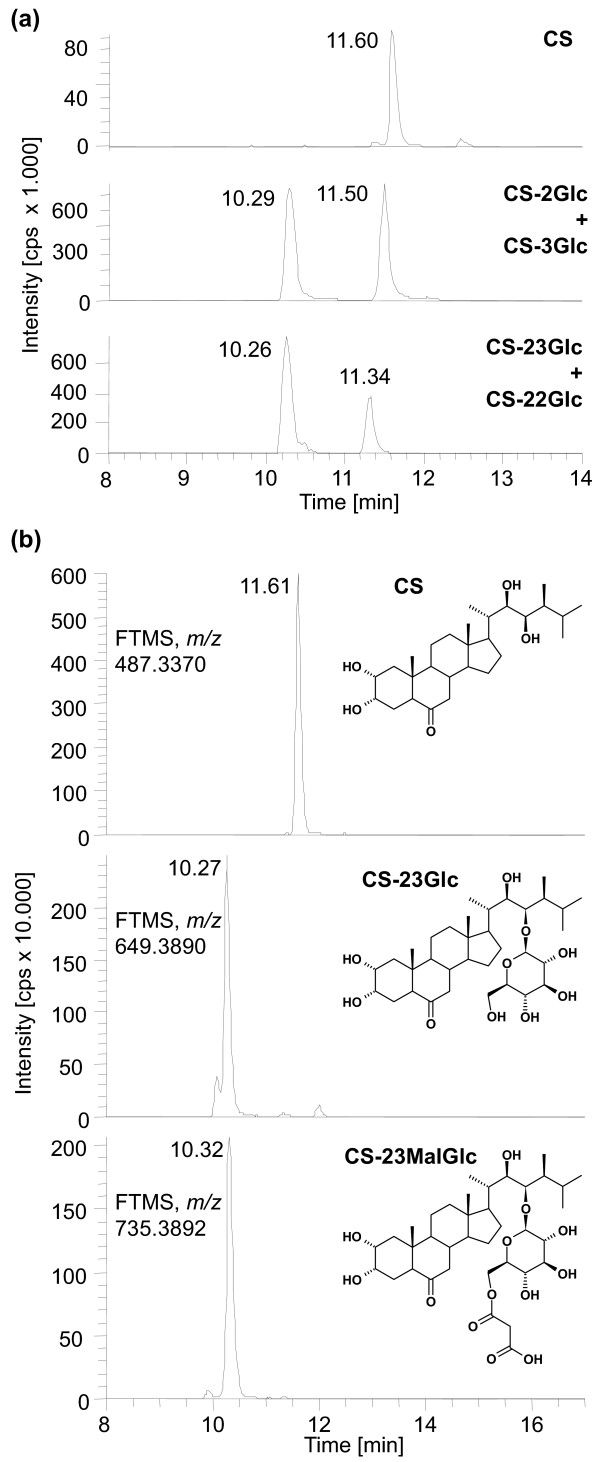
**Identification of a novel CS-Glc catabolite**. HR-LCMS analysis was employed to identify glucosides formed in BL feeding experiments of seedlings over-expressing *UGT73C5*. (a) HR-LCMS mass chromatograms of authentic CS-*O*-glucoside standards. (b) HR-LCMS mass chromatograms of metabolites formed in *UGT73C5oe *seedlings. Theoretical masses of sodium adducts and predicted structures are shown. Please note that the position of the malonyl group in the putative CS-23MalGlc is not certain.

**Figure 8:**
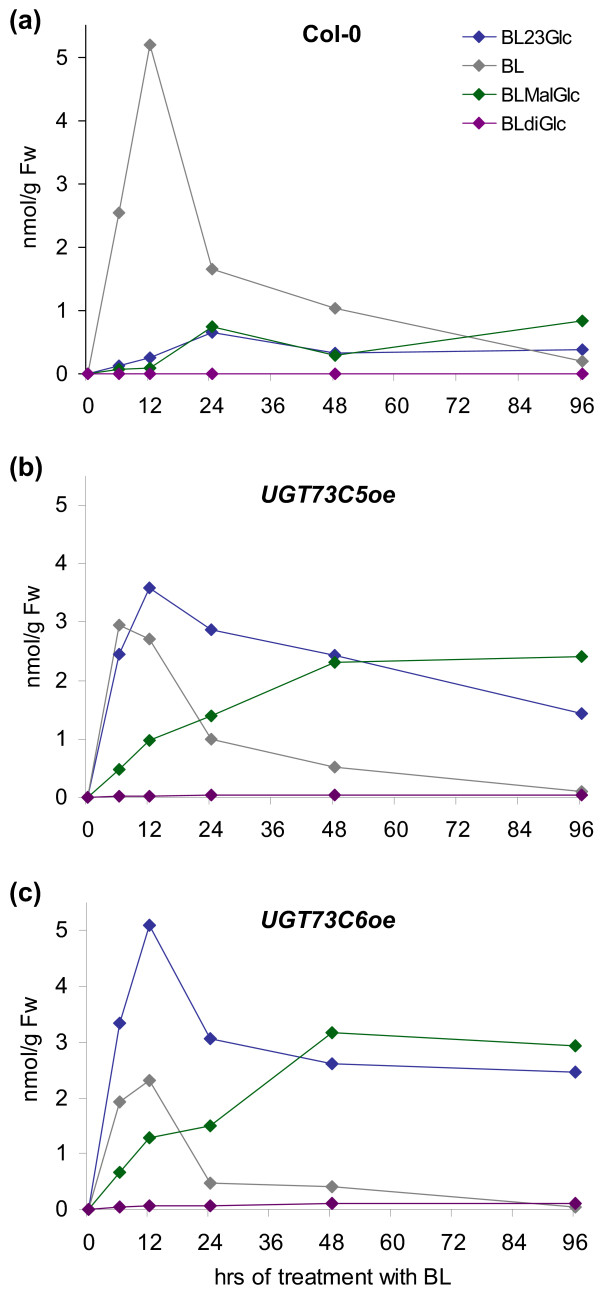
**Analysis of wild type and *UGT73C5oe *and *UGT73C6oe *seedlings, used in BL feeding studies, for the occurrence of BL-MalGlc over time**. The values shown are nmol/g Fw.

Similarly, as shown in Figure 7, when plants fed with CS were analyzed for CS-catabolites a peak at 10.29 min (compared to 10.25 min of CS-23G), showing a *m/z *of 735.3929, appeared and was tentatively identified as CS-malonylglucoside (CS-MalGlc). Only one sum formula is conceivable when applying the criteria outlined above, namely C_37_H_60_O_13 _(mass deviation 0.4 ppm). Again, it seems highly likely that the substance is CS-23-*O*-malonylglucoside (CS-23MalGlc). In addition to the putative BR-MalGlcs BR-diglucosides (BR-diGlc) were also identified. Interestingly, both the formation of the putative BR-MalGlcs and the BR-diglucoside was increased in *UGT73C6oe *and *UGT73C5oe *seedlings as compared to those of wild type indicating that they were formed from BR-23Glcs (Figure 8). In analogy to BR-23Glc both BR-diGlc and the putative BR-MalGlc were not only detected in plant extracts, but were also present in the media in which plants had been incubated for the feeding studies (data not shown); thus BR-Glcs formed *in planta *were released to the media.

In summary these results suggest that 23-*O*-glucosides of BL and CS are further modified by malonylation *in planta*.

### Kinetics of BL-glucoside catabolism in *UGT73C5oe *and *UGT73C6oe *plants

To determine the kinetics of formation of the putative BR-MalGlc and BR-diGlc, the samples of the time-course BL feeding studies were analyzed for an occurrence of BL-23Glc catabolites. At present no analytical standard is available for BL-MalGlc to accurately quantify its amounts. However, as a rough estimate the same response factor as for BL-23Glc was assumed, allowing for a semi-quantitative estimation of BL-MalGlc concentrations. Similarly the concentration of BL-diGlc was estimated by assuming the same response factor as for BR-23Glc. The results are illustrated in Figure 8 and show levels of BL-MalGlc and BL-diGlc in nmol/g Fw, as compared to BL and BL-23Glc amounts in seedlings of Col-0, *UGT73C5oe *and *UGT73C6oe*. All BL-Glcs detected were present in all analyzed lines, however in wild type BL-diGlc was close to the limit of detection with the applied LC-HRMS method. Amounts of the putative BL-MalGlc increased in wild type for 12 hrs and were then sustained (Figure 8a). Similarly the kinetics of putative BL-MalGlc formation in *UGT73C5oe *and *UGT73C6oe *lines were characterized by an increase to a plateau concentration within 48 hrs of feeding, which was then sustained for the rest of the experiment (Figure 8b, c). This is in contrast to BL and BL-23Glc levels, which decreased in both wild type and *UGT73C5oe *and *UGT73C6oe *after having reached a peak. Interestingly, a drop in BL-23Glc amounts correlated with a corresponding increase in putative BL-MalGlc in *UGT73C5oe *and *UGT73C6oe *plants, supporting the notion that BL-23Glc was converted to BL-23MalGlc.

In summary the results show that in BL feeding studies of wild type, and *UGT73C5oe *and *UGT73C6oe *plants a decrease in BL-23Glc levels correlated with an increase in putative BL-MalGlc, showing that BL-23Glc was further conjugated. As opposed to BL and BL-23Glc the putative BL-MalGlc did not decrease after an initial increase, suggesting that malonylation may protect 23-*O*-glucosylated BL from removal, in the soluble fractions analyzed.

## Discussion

Glycosylation is considered an important regulatory mechanism that contributes to the control of hormone homeostasis and almost all major classes of hormones occur as glycoside-conjugates *in planta *[10,11,21]. BRs are one class of plant hormones, which are glycosylated [2,7] and previously we have shown that conjugation to glucose reduces BR activity. Over-expression of *UGT73C5 *led to a massive increase in BR-23-*O*-glucosylation activity and to decreased levels of bioactive BRs, evidenced both at the chemotypic and at the phenotypic level [8]. *UGT73C5 *belongs to a cluster of six closely related genes in the *A. thaliana *genome, *UGT73C1-C6 *[13]. The *in vitro *catalytic activities of the six gene products have been characterized to some extend, and it appears that members of the UGT73C subfamily can recognize a number of aglycons including secondary metabolites, plant hormones, fungal mycotoxins and xenobiotics as substrates *in vitro *[12,15,16,18,20,22]. However, nothing was known of the consequences of over-expressing the five remaining members of the 73C gene cluster *UGT73C1-C4 *and *UGT73C6 *on plant growth and development and in particular also on those growth processes regulated by BRs. This study was designed to investigate those consequences, aiming at identifying possible functional homologues of UGT73C5, and revealed that UGT73C6, the closest homologue of UGT73C5, can also accept BRs as substrates *in planta*.

Overexpression of *UGT73C6 *led to the same phenotypic effects as observed in *UGT73C5oe *plants: growth defects indicative of BR deficiency. Moreover at the chemotypic level *UGT73C6oe *plants were characterized by significantly reduced amounts of TY, 6-deoxoCS and CS, which correlated with a strongly increased 23-*O*-glucosylation activity in CS and BL feeding studies. These data show that *in planta *UGT73C6 can catalyze 23-*O*-glucosylation of CS and BL and is likely also able to glucosylate TY and 6-deoxoCS. Interestingly, UGT73C6 was previously identified as a flavonol-3-*O*-glycoside-7-*O*-glucosyltransferase [14] and was *in vitro *capable of recognizing an array of structurally highly diverse aglycons [12,14-16]. Thus the question arose if, in addition to its role in quercetin-3-*O*-rhamnoside conjugation, UGT73C6 may also catalyze BR glucosylation *in planta*. To try to answer this question seedlings of a *UGT73C6ko *line were analyzed for alterations in BR-23-*O*-glucosylation activities, but no significant change in the formation of CS- and BL-23Glcs were found. This result can be interpreted in several ways. First, it is possible that the endogenous gene *UGT73C6 *does not function in BR-23-*O*-glucosylation *in planta*. Second, the expression and function of UGT73C6 may be highly specific to particular cells or developmental events, and the impact of losing its activity was not observed under the conditions assayed in this study. Third, UGT73C5 or other GTs that glucosylate BRs *in planta*, and are co-ordinately regulated, may complement for a loss of UGT73C6 function and thus, knocking out *UGT73C6 *only will not produce a phenotype. Indeed functional redundancy is characteristic of regulatory events governing BR action [4,23] and has also been shown to play a role in BR catabolism: the cytochrome P450 monooxygenase SOB7 acts redundantly with BAS1 in the inactivation of BRs [24]. To refine UGT73C6 function in the context of functional redundancy it was therefore aimed to generate plants deficient in the expression of both *UGT73C6 *and *UGT73C5*. However, several approaches including the use of RNAi [25] and artificial microRNAs [26], failed in generating double knock-down plants. Thus, in summary we provide evidence that UGT73C6 is capable of glucosylating BRs *in planta*, however at present we cannot answer the question if BR glucosylation is also a physiological function of UGT73C6. Further work will be needed to address this issue.

The expression of *UGT73C6 *was analyzed at the subcellular and cellular level and it was found that *UGT73C6 *shows over-lapping, but also distinct expression/localization patterns with *UGT73C5*. GUS reporter data suggests that transcript abundance of both genes is developmentally regulated and is enriched in vascular tissues, which are also tissues in which genes involved in BR biosynthesis are preferentially expressed [27,28]. At the transcriptional level, in seedlings, *UGT73C6 *expression was not found to be responsive to externally applied BR. However, interestingly *UGT73C6 *mRNA levels are increased in response to a large variety of stimuli including (a) toxins of exogenous and endogenous origin such as the mycotoxin deoxynivalenol [20], the explosive TNT [16], the herbicide imidazolinone [29], as well as the allelochemical benzoxazolin-2(3*H*)-one [30] and oligogalacturonides released from plant cell walls by pathogen polygalacturonases [31], and (b) abiotic and biotic stress factors such as salt stress [32,33] and *Botrytis cinerea *infections [34]. Therefore UGT73C6 has been proposed to comprise a component of a co-ordinately regulated, broad specificity, xenobiotic defense response machinery [30]. *UGT73C5 *shows a similar responsiveness to toxins in its transcriptional regulation [20] and it will thus be interesting to determine how responsiveness to abiotic and biotic stimuli is coordinated with the putative functions of *UGT73C5 *and *UGT73C6 *in glucosylating BRs and/or flavonols.

On a cellular level YFP reporter data indicate that UGT73C5 and UGT73C6 localize to the cytoplasm and intriguingly also to the nucleus. In mammals, where there is considerable interest in UDP-glucuronyltransferases as regulators of metabolic homeostasis, it is thought that, in addition to cytoplasmatic functions, UGTs may also act in the nucleus to control the steady state of ligands for nuclear receptors and protect nuclear components from toxins [35,36]. In this context the UGT2B7, which glycosylates steroid hormones, retinoids, fatty acids as well as xenobiotics, has been shown to be present and active both in the ER and in the nucleus [37]. Also plant UGTs have previously been found to exhibit dual subcellular localizations [38], however the functional significance is as yet unknown.

Another so far unresolved question is the function of BR-Glc formation. Whereas it is well documented that glycosylation can alter the bioactivity of plant hormones including auxins, cytokinins, abscisic acid and gibberellins the reason why glycoside conjugates are inactive is unclear [39,40]. In principle glycosylation could inhibit hormone activity directly by interfering with receptor recognition or indirectly by inducing events, which are enabled by the glycosylation status [40]. In this context, glycosylation is known to facilitate transport and results of this study indicate that also BR glycosides are transported, either actively or passively. Glycosylation is also considered to alter the stability of aglycons [41] and here first evidence is presented which indicates that 23-*O*-glucosylation protects BRs from degradation and/or catabolism. Moreover it is shown for the first time that CS- and BL-23Glcs are further conjugated, likely by malonylation.

Malonylation is an aliphatic acylation, which involves a regiospecific malonyl group transfer from malonyl-CoA to the glycosyl moiety of a glycoside, and is catalyzed by acyltransferases of the BAHD family [42,43]. Malonylation modifies secondary metabolites such as flavonoids, isoflavonoids, anthocyanins and terpenoids and is considered to enhance solubility, protect glycosides from enzymatic degradation by glycosidases and facilitate their intracellular transport [42,43]. Malonylation has also been implicated in the regulation of hormone homeostasis. The ethylene precursor ACC can be irreversibly conjugated to form *N*-malonyl-ACC [44] and thus malonylation of ACC decreases the levels of ethylene in producing tissues. In regard to BR catabolism the results of this study show that the putative BL-MalGlc formed in *UGT73C5oe *and *UGT73C6oe *lines is less readily removed from soluble cellular fractions than BL-23Glc, indicating that malonylation may serve to protect BL-23Glc from catabolism or degradation by enzymes such as glucosidases. In this context it will be interesting to determine if de-glucosylation is a means of reactivating BRs from BR-Glcs and thus, if BR-Glcs may serve as readily available BR storage forms.

## Conclusions

In summary this study provides evidence that in addition to UGT73C5, also its closest homologue UGT73C6, is able to catalyze 23-*O*-glucosylation of the bioactive BRs CS and BL *in planta*. Future studies will address the question, if BR glucosylation is a physiological role of both UGTs, and if this potential multiplicity may provide a highly flexible system for homeostatic adaptation at a cellular level.

## Methods

### Plant material and growth conditions

*A. thaliana *ecotype Columbia-0 (Col-0) was used as the wild type for all experiments carried out in this study. For phenotypic analysis, if not indicated differently, plants were cultivated in a growth room with long-day growth conditions (16 hrs white light, 80-100 μmol·m^-2^·s^-1^/8 hrs dark) at 21 ± 2°C. Plant transformation and seed sterilization was performed as described previously [45]. ATS media [46] was used for plant growth under sterile conditions.

### Chemicals

BL and CS were purchased from Synthchem Inc. (Waterloo, Ontario, Canada). 24-epiBL was obtained from Sigma-Aldrich (St. Louis, USA). Stock solutions of 100 μg/mL in ethanol were made and stored in amber screw vials at -20°C. Synthesis of 2-*O*-, 3-*O*-, 22-*O*- and 23-*O*-Glcs of BL and CS will be described elsewhere (Seto, unpublished). The BR-Glcs were stored in amber screw vials at -20°C as 50 μg/mL stocks in ethanol. Water for LC was purified using a MilliQ system (Millipore, Molsheim, France). LC gradient grade methanol, acetonitrile and sodium chloride (p.a.) were purchased from Merck (Merck KGaA, Darmstadt, Germany). LC-MS grade formic acid was obtained from Sigma-Aldrich (St. Louis, USA). Ethyl acetate was supplied by Carl Roth (Karlsruhe, Germany). Strata Si-1 silica gel SPE cartridges (500 mg, 6 mL) and security guard C18 precolumns were acquired from Phenomenex (Aschaffenburg, Germany).

### Generation of transgenic lines

For the generation of plants over-expressing individual members of the UGT73C subfamily, the open reading frames of *UGT73C1, UGT73C3, UGT73C4 *and *UGT73C6 *were PCR amplified from plasmids containing the corresponding genes [13] and were cloned into a modified version of the binary vector pBIN19 called pJR1Ri, in which expression of the transgenes is driven by the *CaMV35 S *promoter [47]. *UGT73C2 *was amplified from genomic DNA with the primer pairs UGT73C2-fw/UGT73C2-rv (for sequences of all primers used see Additional File 1) and was cloned *EcoR*V and *Not*I into the binary plant expression vector, pGWR8, which also expresses transgenes under control of the *CaMV35 S *promoter [48].

Plants of *A. thaliana *were transformed with the representative constructs using the floral dip method [45] and 15-25 independent transgenic lines were selected for each construct. Plants homozygous for the transgenes were then analyzed by semi-quantitative RT-PCR analysis for transcript abundance using gene specific primers and 2-5 lines with high expression levels were chosen for phenotypic analysis.

For the generation of *UGT73C6 *promoter GUS lines the promoter and the 5' UTR of *UGT73C6 *(-1687 to +4 relative to the translational start) was PCR amplified from genomic *A. thaliana *DNA using Taq polymerase (Fermentas, St. Leon-Rot, Germany) and the primer pair 73C6p-GUS-fw/73C6p-GUS-rv, and was cloned into pPZP-GUS.1 using *Pst*I*+Bam*HI [20]. Plants were transformed with the constructs, 20 independent lines were selected and a line with a representative GUS expression pattern was chosen for subsequent analysis.

For the generation of YFP reporter lines the ORFs of *UGT73C5 *and *UGT73C6 *were PCR amplified from genomic *A. thaliana *DNA using Taq polymerase and the primers 73C5cds-YFP-fw/73C5cds-YFP-rv and 73C6cds-YFP-fw/73C6cds-YFP-rv, and the PCR products obtained were cloned *Nco*I*+Not*I into pGWR8 [48] down-stream of the *CaMV35 S *promoter. YFP was then added in frame to the C-terminal parts of the genes to create YFP-fusion constructs. A YFP reporter construct driven by the endogenous UGT73C6 promoter was cloned by PCR, amplifying the 5'UTRs of *UGT73C6 *from genomic DNA using primers 73C6p-YFP-fw/73C6-YFP-rv and replacing the 35 S promoters with the obtained PCR product. Following plant transformation 10-20 independent transgenic lines were selected for each construct and plants homozygous for the transgenes were analyzed by Western blotting for YFP fusion protein abundance.

### Analysis of BR levels using gas chromatography mass spectrometry (GC-MS)

For BR measurements plants were grown in long-day conditions (16 hrs cool white light, 80-100 μmol·m^-2^·s^-1^/8 hrs dark) at 21 ± 2°C for 24 days before tissue of aerial plant parts was harvested. Fifty grams (fresh weight) of plant material was lyophilized and extracted twice with 500 ml of MeOH:CHCl_3 _(4:1) and deuterium-labeled internal standards 1 ng/g fresh weight were added. Purification and quantification of BRs was performed as described previously [49].

### Sample preparation for the analysis of metabolism of BL and CS in plants

Fifty eleven-day-old seedlings of Col-0, *UGT73C6oe*, *UGT73C6ko *and *UGT73C5oe*, grown on ATS plates, were transferred to sterile Erlenmeyer flasks containing 30 ml liquid ATS media and incubated on a shaker (60 rpm) in continuous light (80 μmol·m^-2^·s^-1^) conditions at 21°C ± 2°. 24 hrs after transfer of the plants, BL or CS were added to an end concentration of 1 μg/ml (2.1 and 2.2 μM, respectively) and the seedlings were incubated for the indicated periods of time. The plant material (on average 0.8 to 1.0 g) was then harvested, ground in liquid nitrogen and extracted twice with 5 ml aqueous methanol (50+50, v+v). The methanolic plant extracts (10 ml) were dried down under a gentle stream of nitrogen at 40°C and re-dissolved in 2.5 ml saturated NaCl solution and 2.5 ml water. Liquid/liquid extraction was performed 3 times with 5 ml ethyl acetate each. The ethyl acetate phases were combined, dried down under nitrogen and re-dissolved in 1 ml ethyl acetate. Strata Si-1 silica gel SPE cartridges were conditioned with 5 ml acetonitrile and equilibrated with 10 ml ethyl acetate before the sample was applied. The cartridges were washed with 5 mL ethyl acetate, removing most of the chlorophyll. The BRs and their glucosides were eluted with 5 ml of ethyl acetate/methanol 20/80 (v/v), dried under nitrogen and reconstituted in 1 ml 70% methanol for analysis by LC-HRMS. For the time course experiment samples were taken before and 6, 12, 24, 48 and 96 hrs after addition of BL (1 μg/ml).

### Liquid chromatography high-resolution mass spectrometry (LC-HRMS) for the analysis of BRs and their glucosides

A LC-HRMS method was developed for the quantification of BRs and BR-Glcs as well as their metabolites produced in plant tissues. An Accela HPLC pump (Thermo Fisher Scientific, Waltham, MA, USA) together with a Mistral column thermostat (Maylab, Thermo Fisher Scientific) and a PAL HTC autosampler (CTC Analytics, Zwingen, Switzerland) were coupled to a LTQ Orbitrap XL high-resolution mass spectrometer (Thermo Fisher Scientific). Separation was performed on a Hypersil Gold column (150 × 2.1 mm, 3 μm particle size; Thermo Fisher Scientific) at 25°C. Gradient separation used water with 0.1% formic acid as solvent A and methanol with 0.1% formic acid as solvent B. 50% B were kept for 1 min, then a linear gradient reached 100% B at 12 min. After a 5 min washing step with 100% B, the solvent composition was changed back to 50% B within one min and the column was re-equilibrated till the end of the run at 32 min. A divert valve redirected the eluent into the ion source between 8 and 13 min to minimize unnecessary contamination of the MS. A flow rate of 250 μl/min was chosen, the injection volume was 5 μl. Ionization was performed in the electrospray positive mode at 300°C with the following settings: sheath gas flow 45, aux. gas flow 5, source voltage 4 kV, capillary voltage 5 V, tube lens 200 V. Centroid FTMS data were acquired from *m/z *200-1000 with a resolution of 60.000. The sodium adduct of n-butylbenzenesulfonamide (nBBS; *m/z *236.071570) was found to be ubiquitous in our system and was used as lock mass. Instrument control and data evaluation was performed with Xcalibur 2.0.7. For the latter, a mass tolerance of 5 ppm was allowed for the following masses: [BL+Na]^+ ^*m/z *503.3343; [CS+Na]^+ ^*m/z *487.3394; [BL-glucosides+Na]^+ ^*m/z *665.3871; [CS-glucosides+Na]^+ ^*m/z *649.3922. Retention times were: BL23G: 9.54 min; BL2G: 9.74 min; CS23G: 10.25 min; CS2G: 10.32 min; BL22G: 10.72 min; BL3G: 10.79 min; BL: 10.97 min; CS22G: 11.34 min; CS3G 11.50 min; CS 11.60 min.

External standard calibration was performed with 1/x weighted models. While the parent substances BL and CS showed highly linear correlations from 1 - 1000 ng/ml, quadratic models were used for all eight glucosides over the same concentration range. Recovery and repeatability was tested by spiking 100 ng/ml of all analytes in liquid media or methanolic extracts of untreated plants in quadruplicates before clean-up and measurement.

### Semi-quantitative and quantitative real-time PCRs

For semi-quantitative RT-PCRs RNA was isolated from plant material using the RNeasy Plant Mini Kit from Qiagen (Qiagen GmbH, Hilden, Germany) and cDNAs were synthesized with the RevertAid H Minus First Strand cDNA Synthesis Kit (Fermentas) from DNaseI-treated RNA. PCRs were performed with specific primers for the *UGTs 73C1, 73C2, 73C3 *and *73C4 *(sequences see Additional File 1) and *UGT73C5 *[20] and *UGT73C6 *[8].

For analysis of transcript levels by qPCR five-day-old seedlings, grown on ATS plates, were transferred to sterile Erlenmeyer flasks containing 30 ml of liquid ATS media and incubated on a shaker (60 rpm) in continuous light (80 μmol·m^-2^·s^-1^) at 21°C ± 2° for 3 d. Subsequently, 24-epiBL (dissolved in DMSO) was added to an end concentration of 1 μM and the plants were incubated for another 24 hrs under the same conditions. As a control, seedlings were treated with an equal amount of DMSO. RNA isolation, cDNA sythesis and qPCRs were performed as described previously [50] using the primers listed in Additional File 1. The relative expression levels were calculated from three biological replicates, each measured in technical quadruplicate, after normalization to *GAPC2 *[51]. The expression levels of treated and untreated samples were considered as statistically significantly different, if the p-value of a two-tailed t-test using log_2_-transformed results was below 0.05.

### Western blot analysis

Leaf tissues (100 mg) were ground in liquid nitrogen using a Qiagen TissueLyser II and extracted with 300 μl extraction buffer (66 mM TRIS/HCl pH 6.8, 133 mM DTT, 2.7% SDS, 13% glycerol, 0.01% bromophenol blue). 20 μl of these extracts were separated by SDS-PAGE (10% gel) and blotted onto Immobilon P (Millipore Cooperation, Bedford, MA, USA). The membranes were first incubated with mouse anti-GFP antibody and second with alkaline phosphatase-conjugated goat anti-mouse IgG (Santa Cruz Biotechnology, CA, USA). Detection was performed by enhanced chemiluminescence using the CDP-Star detection reagent (Amersham Bioscience, NJ, USA).

### Reporter localization analysis

GUS activity of *UGT73C6_pro_:GUS *lines was analyzed histochemically as described previously [20]. For YFP localization studies seedlings were grown on ATS plates [46] for 11 days and were subsequently analyzed with a Zeiss LSM Meta confocal microscope (Zeiss, Oberkochen, Germany). YFP-tagged fusion proteins and chlorplast autofluorescence were both excited using the Ar laser line at 488 nm and were detected at 530-565 nm (yellow channel) and 625-700 nm (red channel), respectively. The images were assembled using the Zeiss LSM image browser software version 4.2.0.121. For co-localization studies YFP and CFP-tagged versions of UGT73C6 and BES1 [48] were transiently expressed in leaves of *Nicotiana bentamiana *by infiltration with agrobacteria as described previously [52]. Infiltrated leaves were examined by fluorescence microscopy using a Zeiss Axioplan 2 microscope equipped with a Zeiss AxioCam MRc5 camera.

## Authors' contributions

SH carried out the GUS reporter analysis, generated and analyzed YFP reporter lines, generated and analyzed over-expression lines, performed the BR feeding studies and helped to draft the manuscript. FB developed the LC-HRMS method, performed the BR glucoside analyses and helped to draft the manuscript. SF carried out the analysis of endogenous BR contents. WR performed co-localisation experiments, participated in the analysis of the BR glucoside formation data and supported the coordination of this study. MK and FK performed expression analyses. LE, GSH and YL participated in the generation of over-expression lines. RS, RK and FEV participated in the coordination of this study. HS synthesized the BR-glucosides. DB participated in the design and coordination of this study and helped to draft the manuscript. BP conceived the study, participated in its design and coordination, wrote the manuscript and performed experimental work, such as the generation and analysis of over-expression and GUS reporter lines. All authors read and approved the final manuscript.

## Acknowledgments and Funding

We would like to thank the horticultural staff of the University of York and the Max F. Perutz Laboratories for excellent plant care and Prof. Kazuki Saito for kindly providing seeds of the *UGT73C6ko *line. We also thank Dr. Suguru Takatsuto (Joetsu University of Education) for supplying deuterium-labeled internal standards. This work was supported by funds from the Austrian Science Fund FWF, the United Kingdom Biotechnology and Biological Sciences Research Council, the Garfield Western Foundation and by a Grant-in-Aid for Scientific Research (B) from the Ministry of Education, Culture, Sports, Science and Technology of Japan to SF (Grant No. 19380069). The LC-MS system was funded by the Federal Country Lower Austria and co-financed by the European regional development fund of the European Union. MK received a fellowship from the Higher Education Commission of Pakistan; BP received an Erwin-Schrödinger and a Hertha-Firnberg fellowship from the FWF.

## Supplementary Material

Additional file 1 **Primers used in this study**. A table providing the sequences of primers used in this study. Click here for file
