# Supplementary material for: Overexpression of the UGT73C6 alters brassinosteroid glucoside formation in Arabidopsis thaliana
Source: BMC Plant Biol. 2011 Mar 24;11:51. doi: 10.1186/1471-2229-11-51 (PMC3073898; doi:10.1186/1471-2229-11-51)
Supplement: Additional file 1 — Primers used in this study. A table providing the sequences of primers used in this study. [file 1471-2229-11-51-S1.PDF]

Supporting Table 1

Name		Sequence	
73C1RT-fw	5’	TGAGAAATTGGCGGTGCAG	3’
73C1RT-rv	5’	TCATTTCTTGGGTTGTTCTA	3’
73C2RT-fw	5’	GGAGAGTATTGGAGTGTTA	3’
73C2RT-rv	5’	TCAACTCTTGGATTCTACTT	3’
73C3RT-fw	5’	TGGAAGAATTGATGGGTGAT	3’
73C3RT-rv	5’	TCAATTCTTGAATTGTGCTA	3’
73C4RT-fw	5’	GGAGAAAATAGGAGTGTTA	3’
73C4RT-rv	5’	TCAGTTCTTGGATTTCACT	3’
73C5cds-YFP-fw	5’	ATAAGTACTATGGTTTCCGAAACAACCAAATC	3’
73C5cds-YFP-rv	5’	ATAGCGGCCGCAATTATTGGGTTCTGCCAGTTCC	3’
73C6cds-YFP-fw	5’	AGTTGGATATCCATGGCTTTCGAAAAAAAC	3’
73C6cds-YFP-rv	5’	ATATGCGGCCGCAATTATTGGACTGTGCTAGTTG	3’
73C6p-GUS-fw	5’	ACCTGCAGAACCCAATAATTGAGTATACGTC	3’
73C6p-GUS-rv	5’	TTCGGATCCCATGACGATGCAACTTTAGTA	3’
73C6p-YFP-fw	5’	CACATTTGTACGAATTAGCAGAAGAC	3’
73C6p-YFP-rv	5’	CCATGGATATCCAACTTTAGTAAGAACC	3’
BR6Ox2qPCR-fwd	5’	AGCTTGTTGTGGGAACTCTATCGG	3’
BR6Ox2qPCR-rev	5’	CGATGTTGTTTCTTGCTTGGACTC	3’
GAPC2qPCR-fwd	5’	TTGGTGACAACAGGTCAAGCA	3’
GAPC2qPCR-rev	5’	AAACTTGTCGCTCAATGCAATC	3’
UGT73C2-fw	5’	TAGAGATAGGATATCATGGC	3’
UGT73C2-rv	5’	AACAATATAGCGGCCGCAAC	3’
UGT73C5qPCR-fwd	5’	AAGATCCGGGGTTGAACAGC	3’
UGT73C6qPCR-fwd	5’	GTAAGTGCCGAGGTTAAAGAGG	3’
UGT73C5/6qPCR-rev	5’	TCTCCAAGCTCTTTGGCTCT	3’
